# The Interplay between COMT and MIR146a Supports the Role of Immunity in Alzheimer’s Disease

**DOI:** 10.1093/geroni/igaf122.3191

**Published:** 2025-12-31

**Authors:** Anatoliy Yashin, Deqing Wu, Konstantin Arbeev, Olivia Bagley, Hongzhe Duan, Rachel Holmes, Svetlana Ukraintseva

**Affiliations:** Duke University, Durham, North Carolina, United States; Duke University, Durham, North Carolina, United States; Duke University, Durham, North Carolina, United States; Duke University, Durham, North Carolina, United States; Duke University, Durham, North Carolina, United States; Duke University, Durham, North Carolina, United States; Duke University, Durham, North Carolina, United States

## Abstract

Accumulating evidence points to a major role of compromised immunity in Alzheimer’s Disease (AD). The MIR146a and COMT genes are known for their involvement in both immune responses and AD. COMT also regulates the metabolism of catecholamines, which are linked to inflammation and trained immunity. We hypothesize that the interplay between MIR146a and COMT genes influences the risk of AD. To test this hypothesis, the logistic regression model with the interaction term has been applied to the analysis of the Cardiovascular Health Study (CHS) data on males and females combined. We used the binary AD trait with cases (coded as 1) including participants who survived age 85 and did not have AD before or after this age (N = 1,421) and controls (coded as 0) including participants who acquired AD at any age (N = 257). We found that the interaction between SNPs rs2910163 in MIR146a and rs4818 in COMT is significantly associated with AD (β=-0.48, p = 1.62E-02, Bonferroni=1.67E-02). This result for the same SNPs was confirmed in the Framingham Heart Study (FHS) data analysis, with case and control groups including 903 and 354 participants, respectively (β=-0.41, p = 3.63E-02). The SNP rs2910163 is in high linkage disequilibrium (LD) with rs2910164, D’=1, R2=0.77. In other studies, SNPs rs4818 and rs2910164 (studied separately) have been associated with multiple health-related traits. The results of this study support the role of immunity in AD and emphasize the importance of studying the roles GxG interactions in AD.

